# Female chronotype relates to lay date but not fitness in an island population of great tits

**DOI:** 10.1007/s00442-025-05857-3

**Published:** 2026-01-10

**Authors:** Aurelia F. T. Strauß, Barbara M. Tomotani, Barbara Helm, Marcel E. Visser

**Affiliations:** 1 Groningen Institute for Evolutionary Life Sciences (GELIFES), University of Groningen, Nijenborgh 7, 9747 AG Groningen, The Netherlands; 2 Department of Animal Ecology, Netherlands Institute of Ecology (NIOO-KNAW), P.O. Box 50, 6700 AB Wageningen, The Netherlands; 3 Department of Arctic and Marine Biology, UiT The Arctic University of Norway, Framstredet 41-42, N-9037 Tromsø, Norway; 4 Bird Migration Unit, Swiss Ornithological Institute, Seerose 1, 6204 Sempach, Switzerland

**Keywords:** Individual differences, Diel timing, Life history, Breeding season, Reproductive success

## Abstract

Organisms use diel timing mechanisms to anticipate predictable daily environmental fluctuations, such as the start and end of the day light period. There is often ample intraspecific variation in this diel timing, with individuals being consistently active earlier or later than others in the population and therefore having early or late chronotypes. In tit species, early-active males have higher rates of extrapair paternity, and early-active females might have more offspring, thereby increasing their fitness. However, studies on these fitness consequences of chronotype tend to be inconclusive, based on small sample sizes and confounded with seasonal effects on activity timing. Here, we measured the fitness of standardised chronotype in female great tits (*Parus major*) across three breeding seasons. We extracted activity onsets, when females first left the nest in the morning, from recordings of nest temperatures during incubation and chick provisioning. To account for seasonal and daily variation in the timing of activity, we expressed these onsets relative to the conspecifics active on the same day. The chronotypes of 164 females were tested for differences in fitness and life-history parameters from brood monitoring data. We show that chronotype was not significantly related to fitness parameters, such as the number of fledglings and hatchlings, nor to offspring and female condition. However, extremely early and late chronotypes started breeding later in our population, but not in the re-analysed datasets from three other populations. Our findings suggest that chronotype is not under strong selection, or perhaps under fluctuating selection, allowing high between-individual variation to persist.

**Supplementary Information:**

The online version contains supplementary material available at 10.1007/s00442-025-05857-3.

## Introduction

Diel rhythmicity is thought to be an adaptive trait (Anderson and Wiens 2017) because it enables the anticipation of predictable situations, such as the start of the day, and this way supports the coordination of physiological and behavioural rhythms (West and Bechtold 2015). Diel timing may be essential for optimising energy acquisition and consumption over the 24-h cycle of the day, to match activities with cycles of environmental conditions such as food availability and predation risk. It is particularly beneficial to synchronise foraging timing with high food abundance (Norberg 1977; McNamara et al. 1994), and to avoid times of high predation pressure and unsuitable abiotic conditions (Kacelnik 1979; van der Vinne et al. 2019). For example, studies have shown shifts in activity patterns of animals in response to hunting or disturbance by humans (Martorell-Barceló et al. 2018; Bonnot et al. 2020). At an intraspecific level, synchronisation of conspecifics may decrease predation risk and enhance probabilities of finding a mate (Duranton and Gaunet 2016; Hau et al. 2017) but may also increase competition (Duranton and Gaunet 2016) and the transmission of diseases and parasites, especially in social groups (Patterson and Ruckstuhl 2013). Thus, there might be fitness consequences not only for diel timing in general but also for being earlier or later relative to conspecifics.

Chronotypes are defined as consistent individual differences in diel timing in relation to a reference point, usually along an early–late continuum (Schwartz et al. 2017). For wild populations, commonly used reference points are sunrise or sunset (Stuber et al. 2015; Meijdam et al. 2022a; Womack et al. 2023a) or the timing of other conspecifics (Steinmeyer et al. 2013; Schlicht et al. 2023). Irrespective of the reference point, chronotype repeatability (i.e. between-individual variation) accounts for up to 13 to 54% of the total phenotypic variation in great tits (*Parus major*) (Stuber et al. 2015; Meijdam et al. 2022a, b; Womack et al. 2023a). These consistent individual differences may be (partially) due to differences in their circadian system (Dominoni et al. 2013; Tomotani et al. 2023 but see Helm and Visser 2010; Tomotani et al. 2024) and associated genetic background (Steinmeyer et al. 2012; Salmela and Weinig 2019 but see Laine et al. 2019), both targets of selection.

In *Paridae* songbirds, the literature suggests a fitness advantage for earlier chronotypes (Poesel et al. 2006; Greives et al. 2015; Womack et al. 2023a; Schlicht et al. 2023). Earlier-active males were shown to increase reproductive success through higher extrapair paternity, likely because of more extrapair mating opportunities (Poesel et al. 2006; Greives et al. 2015; Schlicht et al. 2023). However, earliness of females did not affect extrapair paternity (Schlicht et al. 2014). Associations between chronotypes and other fitness parameters remain unclear: For example, early-active female great tits had more fledglings (Womack et al. 2023a), but this was not observed in starlings (*Sturnus vulgaris*) (Maury et al. 2020). Further, nestling condition was unrelated to female chronotypes in both species (Pagani-Núñez and Senar 2016; Maury et al. 2020; Womack et al. 2023a) and to male chronotypes in great tits despite a higher chick provisioning rate of early males (Pagani-Núñez and Senar 2016). Taken together, the current literature suggests there could be selection for early chronotypes, but the effects are often inconclusive and based on studies with small sample sizes. Thus, we need further evidence for fitness consequences of chronotype from wild populations.

In wild great tits, chronotype can affect fitness (i.e. lifetime reproductive success) via different fitness parameters related to reproductive success and survival: The number of fledglings might be directly influenced by parental care during chick provisioning and incubation. Early-active females could benefit from provisioning chicks in the early morning, when their energy reserves are depleted (Kontogiannis 1967; Haftorn 1989). Then, chicks’ food demands are highest, matching the highest provisioning rates seen in the early morning (Pagani-Núñez and Senar 2013). The investment into chick provisioning could result in increased nestlings’ condition and fledge success. In contrast, late-active females might increase hatch success and nestling condition by incubating eggs and brooding young nestlings longer in the morning when ambient temperatures are low. This strategy could avoid cooling of the eggs below the suggested optimal conditions for embryo development (Webb 1987; Cooper et al. 2005), and hypothermia of nestlings until they start thermoregulating themselves (Mertens 1977; Engert et al. 2024). Furthermore, chronotype might be crucial to increase survival probabilities. While trading off parental care (e.g. incubation) against self-maintenance (Martin 2002; Coe et al. 2015; Nord and Cooper 2020), early-active females might be able to recover their own energy reserves faster in the morning, increasing body condition. Altogether, selection might differ between breeding stages and potentially also between breeding and non-breeding phases, leading to antagonistic selection pressures. In that case, females with intermediate chronotypes will have the highest fitness by balancing benefits and costs.

Fitness consequences could arise indirectly via different life-history strategies, i.e. fast reproduction *versus* long lifespan (Stearns 1989; Lemaître et al. 2015). When facing limited resources in the wild, individuals might invest less into current and more into future reproduction (Réale et al. 2010; Lemaître et al. 2015). In birds, common life-history traits are clutch size, initiation of second broods, and timing of breeding (i.e. lay date of the first egg), which have been previously linked to fitness (Perrins and McCleery 1989; Verhulst et al. 1997; Visser and Verboven 1999). If these life-history traits are linked to chronotype, they have a great potential to explain fitness consequences of chronotypes. For example, in starlings, early-active females were more likely to initiate a second brood, potentially increasing annual reproductive output or compensating for a lower success in the first brood (Maury et al. 2020). Furthermore, having chicks early in the season is often associated with a higher fitness for the parents (Verboven and Visser 1998). Thus, indirect fitness benefits may arise if there are correlations between parental chronotype and seasonal timing of reproduction. A link between daily activity and breeding timing has already been observed in the wild, whereby earlier chronotypes bred earlier in the season (Murphy et al. 2008; Graham et al. 2017b; but see Steinmeyer et al. 2013; Maury et al. 2020; Meijdam et al. 2022b). This link between annual and diel timing could be mediated through the circadian clock via potentially shared genetics (Le Clercq et al. 2023; but see Verhagen et al. 2019), but evidence for such a mechanism is lacking (Helm 2021).

Measuring individuals’ chronotypes under natural conditions comes with methodological challenges due to phenotypic plasticity in diel timing: First, there are strong seasonal shifts in activity patterns of birds, especially during the breeding season. When days become longer, birds get active earlier relative to midnight (e.g. Womack et al. 2023a). Most studies account for this seasonal shift by using activity timing relative to sunrise or sunset instead of clock time. However, this poses a problem as birds generally advance their activity onset to a lesser extent than the time of sunrise (Schlicht and Kempenaers 2020; Meijdam et al. 2022b; Womack et al. 2023a). Meijdam et al. (2022b) discuss the subsequent bias in measuring chronotype in early- and late-breeding individuals: An early-breeding female is measured under shorter day lengths and thus determined as having an earlier chronotype (relative to sunrise) than a late-breeding female that incubates under longer day lengths (Meijdam et al. 2022b; Tomotani et al. 2023). This bias confounds the comparison of chronotypes and their effects on breeding timing (Meijdam et al. 2022b). Moreover, if there are fitness consequences of breeding time (e.g. higher fitness for earlier breeders (Verboven and Visser 1998)), the bias will lead to an erroneous correlation between chronotype and fitness. Second, day-to-day variation, e.g. in weather conditions, may also affect diel timing (Bermúdez-Cuamatzin et al. 2020; Schlicht and Kempenaers 2020) and could, thereby, further distort the comparison of individuals measured on different days. To counteract these two types of within-individual variation, diel timing can be standardised using measurements of multiple individuals on the same date (Steinmeyer et al. 2013; Schlicht et al. 2023). This method results in a chronotype proxy relative to conspecifics and could be a better alternative to using the onset relative to sunrise because it accounts for shifts in diel timing caused by environmental conditions such as day length and weather (Steinmeyer et al. 2013).

Here, we aim to assess the effects of chronotype on fitness by investigating the annual reproductive success at different breeding stages, adult body condition as well as life-history traits. We monitored activity patterns of female great tits during incubation and chick provisioning, and expressed the onsets of their daily activity in relation to conspecifics at the same date (i.e. first departure from the nest-box in the morning relative to other individuals on that day = standardised onset). We then extracted the chronotype (i.e. the mean standardised onset over all days a female was measured) for each female and linked it to fitness parameters related to reproductive success (i.e. hatch and fledge success, and nestling condition), female condition (i.e. female body weight) and life-history traits (i.e. lay date, clutch size and probability of a second brood). To compare our results with data from other populations, we also re-analysed three published datasets using our standardised chronotype proxy instead of the original onset relative to sunrise (Graham et al. 2017b; Meijdam et al. 2022b; Womack et al. 2023a).

## Material and methods

### Study population and data collection

We studied female great tits in a long-term study population located on a Dutch island in the Wadden Sea (Vlieland: 53.28 N, 5.07 E). Since 1955, great tits have been individually marked and monitored once to twice per week during the breeding season, from nest building until chick fledging (Kluyver 1970). We recorded important avian-specific ecological and life-history data including lay date, clutch size, and nestling number as well as hatch date during more frequent checks (intervals of 1–2 days) around the expected hatch date (on incubation day 14). When nestlings were 7 to 12 days old (hatching date = 0 days old), we ringed all nestlings with a uniquely numbered aluminium ring and caught parents using spring-loaded traps in the nest-box. Adults were individually marked with a uniquely numbered aluminium ring and three colour rings (if unringed), sexed, aged, and biometrics were taken (body weight, tarsus length, and third outermost primary feather (P3) length). When nestlings were 14–16 days old, their biometrics were measured to evaluate the nestlings’ developmental condition and, thus, their probability to recruit as breeders in the next year(s) (Verboven and Visser 1998).

To obtain robust information on possible links between chronotype and fitness, we studied the great tits in three consecutive breeding seasons (April–June; 2020–2022). For comparison of the weather conditions between the three years, we extracted records from close-by weather stations, which revealed substantial weather variation (Royal Netherlands Meterological Institute (KNMI) 2023): Ambient temperature in spring (April–June) 2021 was on average 10.9 °C, and therefore about 1.5 °C colder compared to 2020 (12.2 °C) and 2022 (12.6 °C) (data from the weather station on Vlieland). Furthermore, the weather in May, when most females were incubating or chick provisioning, was cloudier (–173 sun hours) and rainier (+ 125 rain hours and + 121 mm) in 2021 compared to the other two years (i.e. sun: 666 h (2020), 419 h (2021), and 517 h (2022); rain: 18 h & 33 mm (2020), 161 h & 192 mm (2021), and 54 h & 109 mm (2022); average data from weather stations De Kooy and Hoorn (Terschelling)).

We recorded diel activity of female great tits using nest temperature measurements during incubation and chick provisioning. Nest temperatures are widely used for this purpose and have been validated to sufficiently record female activity in great tits (Capilla-Lasheras 2018). When two or more eggs were present, we placed a small temperature logger (iButton: Thermochron DS1922L-F5, Maxim, USA) inside the nest cup recording every three minutes. To avoid removal by the female, the iButton was secured with a metal wire attached to the iButton wrapped in a thin silk sock (2020–2021) or glued to a sewing button (2022). As environmental references for activity data extraction (see below), one to four ambient temperature sensors (HOBO loggers, U12-012, ONSET, USA) were distributed across the study area recording every minute, and then binned and averaged for every 30 min across all loggers.

All procedures were in accordance with the Dutch Experiments on Animals Act and approved by the national Animal Ethics Committee (CCD Permit AVD80100 2017 831).

### Activity onset and chronotype

The nest temperature data allowed us to extract the female’s activity patterns (Capilla-Lasheras 2018; Strauß et al. 2024). Females start incubation by gradually increasing the time spent on the eggs, first at night and later also during the day. When nocturnal full incubation begins, day-night patterns of activity become visible; this incubation start is highly variable between individuals, ranging from 6 days before up to 5 days after clutch completion (Diez‐Méndez et al. 2021). Activity patterns are usually detectable until nestlings develop their own thermoregulation, starting at a body weight of 8 g (ca. at chick day 6) with more stable thermoregulation from chick day 10 (Mertens 1977; Engert et al. 2024). Consequently, nest temperature becomes more homogenous with progressing nestling development, but this might also be nest-specific, depending on the number of nestlings present, their ability to thermoregulate, and the logger’s position within the nest. Following the R pipeline of Strauß et al. (2024), we calculated presence (i.e. on-bouts) and absence (i.e. off-bouts) of the female on the nest. In short, drops and rises of nest temperature were expected to reflect female activity when they exceed the temperature variation observed in the nest during night and day, and in ambient temperature (i.e. nest- and day-specific thresholds; for details see Supplementary Information Sect. 1). The 3-min recording interval allowed us to capture the average great tit off-bout with durations of 7–18 min (Álvarez and Barba 2014) while balancing deployment duration and good accuracy (Capilla-Lasheras 2018). The accuracy of the loggers was set to 0.0625 °C in 2020 and 2021, and to 0.5 °C in 2022 to increase data storage capacity, enabling two weeks of recording without readout. We expect that the different accuracies would not affect onset data as temperature variation was compared within nests between nocturnal and diurnal patterns, and off-bouts occurred with temperature drops > 0.5 °C.

From the female activity patterns, we then extracted the timing of the first off-bout as onset of activity from nocturnal incubation start until nestlings were self-thermoregulating. We included seven activity onsets measured between chick day 10 and 13 in four broods, because these nestlings were in poor conditions and thus allowed us to still record female activity. Nevertheless, it is possible that these onsets might have generated some additional noise. Overall, we obtained 2512 onsets of 264 broods with a median of 10 onsets per brood (range 1–22 days), of which 71% were measured during incubation and the rest (29%) up to 13 days post hatching. Likewise, 62 broods were exclusively measured before hatching and one brood only after hatching.

For the re-analysis of three published datasets (Graham et al. 2017a; Meijdam et al. 2022c; Womack et al. 2023b), we first calculated the activity onset relative to midnight, if not available in the original data. From the Meijdam et al. data, we used the larger dataset with onsets measured during egg laying. From the Womack et al. data, we only included the forest area. Additionally, we estimated lay date using the available incubation start and clutch size (i.e. lay date = incubation start date – clutch size + 1), assuming incubation started on the day of clutch completion to replace the hatch date from the original statistical models and match our own (see below). Using the information about onset, date, and bird IDs, we then proceeded with the standardisation and statistics in the same manner as done for our own data (see directly below).

We standardised the activity onset of a female relative to the activity onset of the conspecifics on the same day, using a z-score, following Steinmeyer et al. (2013) and Schlicht et al. (2023). This way we accounted for environmental variation, especially the effects of day length and weather (see Introduction why this is essential). Specifically, we subtracted the date-specific mean of all females measured on the same date *i* from the raw activity onsets on date *i*, and divided this by the date-specific standard deviation (z-score in standard deviation units):

$${standardised \:onset}_{i}= \frac{\left({femal{e}'s\: onset\: of \:activity}_{i} - {mean \:of\: all\: females}_{i}\right)}{{standard\: deviation\: of\: all \:females}_{i}}$$

In our data, raw activity onsets were mostly distributed around the date-specific mean with a few extremely early exceptions which were equally distributed across individuals, dates, and breeding stages (date-specific SD: mean 0.41 h and range 0.10–0.94 h).

Between-date variation in onsets could come from differences in the set of individuals measured at a given date, including their breeding stage, and from daily environmental variation. Therefore, standardisation is useful for the comparison of individuals measured in different environments (Steinmeyer et al. 2013; Schlicht et al. 2023). To avoid skewed and confounded values in days where a low number of females were recorded, we only included days with at least three females. Similarly, we selected broods with at least two standardised onsets. We obtained repeated measurements (i.e. at least two onsets; n = 2316 onsets) for 184 known females of 232 broods, identified during catching or by observing her colour combination during nest checks, of which 22 females were recorded in two years and 7 in three years. We then determined each female’s chronotype using the mean standardised onset of the first brood in the first year measured for 164 known females (excluding females used in an experiment in Strauß et al. (2024)).

### Fitness and life-history parameters

Female’s fitness was assessed using the overall fledge success (i.e. 0 for none or 1 for at least one fledgling), and the number of fledglings and hatchlings per female of all her broods within the season when she was measured. Thus, each female was included once in our dataset, with chronotype and fitness measured in the same year. The number of fledglings was recorded as the number of nestlings alive at chick day 14–16, excluding nestlings found dead after fledging. The number of hatchlings was determined as the maximum number of nestlings counted during nest checks, plus an uncertainty of hatched nestlings. This uncertainty included the number of hatchlings that were neither observed during the nest checks nor found as unhatched eggs divided by two:

$$number\: of\: hatchling=max. \:observed \:nestlings+uncertainty$$

$$uncertainty=\frac{\left(clutch\: size-max.\: observed \:nestlings-unhatched\: eggs\right)}{2}$$

We further assessed the condition of nestlings (i.e. feather (P3) length, tarsus length, and body weight) and females (i.e. body weight). As life-history traits, we used lay date (in April days, i.e. 1 April is day 1) and clutch size of the first clutch. Lay date was the day of the first egg, which was back-calculated from the first observed number of eggs, assuming females lay one egg per day. We also monitored the initiation of a second brood (i.e. 1 for second brood, 0 for none) after a successful first one (i.e. at least one fledgling), which is particularly common in this population (Verboven and Verhulst 1996).

### Statistics

All data processing and analyses were done using R (version 4.4.0, R Core Team 2024, in the RStudio version 2024.09.0). To assess the consistency of our standardised chronotype, we calculated the repeatability of the onset relative to conspecifics within and across years using the repeated measurements. We fitted linear mixed models with Gaussian error distribution (*lme4* package version 1.1–35.3; Bates et al. 2015) to the standardised onset using independent variables namely *Year*, *April day*, and their interaction (corresponding to date) as well as centred *Breeding day* (i.e. days mean-centred relative to (expected) hatch day) and its quadratic equivalent for the expected non-linearity (Schlicht and Kempenaers 2020). *April day* is highly correlated to time of sunrise, so that the latter was not included in the model. We included random effects namely *Nest-box* and IDs for *Female*, and *Female_Year* to extract repeatabilities for females within and across years (Nakagawa and Schielzeth 2010; Meijdam et al. 2022a).

Fitness and life-history traits were analysed in generalised linear models (GLMs, *lme4* package), including each female once (up to 164 females; data selection criteria and sample sizes in Tables S1–S8). All models included linear and quadratic *Chronotype* as covariates for non-linear effects, *Year* and their interactions with Chronotype for yearly fluctuations in selection, and *Lay date* (centred within year) to account for between-year differences in reproductive timing effects (Jantzen and Visser 2023):

$$response=Chronotype+{Chronotype}^{2}+Year+Chronotype:Year+ {Chronotype}^{2}:Year+ Lay\: date$$

For lay date and size of the first clutch (*n* = 164 females), we fitted Gaussian error distributions, and *Lay date* was excluded as a covariate in the lay date GLM. The numbers of hatchlings or fledglings were analysed using a Gaussian error distribution due to the added uncertainty (see above) and excluded five females who deserted the clutch during the exchange of the iButton (*n* = 159). Fledge success was analysed using a binomial GLM excluding two additional females that failed their brood before the nestlings hatched due to unknown reasons (*n* = 157). Another binomial GLM was fitted to test for differences in the probability of a second brood, in which only females with a successful first brood were included (*n* = 131) while accounting for previous breeding success within the same season by including the *Number of fledglings of the first brood* (Smith et al. 1987). For the Gaussian GLM of female condition (*n* = 153), we used body weight measured during chick provisioning, accounting for linear and quadratic effects of the *Time of day* the weight was taken (Haftorn 1989) and for differences in body size by including *Tarsus size*. For nestling biometrics (weight, tarsus, and P3 feather), we used linear mixed models (LMM) with Gaussian error distributions and additional covariates included: *Hatch date* (centred within year) replaced lay date to account for brood-specific seasonal timing; *Nestling age* (in chick day) and *Number of fledglings* were added as fixed effects (Smith et al. 1987) as well as *BroodID* as a random factor to avoid pseudo-replication of the multiple nestlings measured per brood. For body weight, we accounted again for *Time of day* effects. The data from the three other studies (Graham et al. 2017a; Meijdam et al. 2022c; Womack et al. 2023b) were re-analysed separately in equivalent statistical models. The single-year datasets of Graham et al. and Meijdam et al. excluded *Year* and its interactions with chronotype.

For all models, we used stepwise reduction of parameters and likelihood ratio testing (*anova* function: *test* = *“F”* for Gaussian GLMs and* “Chisq”* for LMMs and binomial GLMs) to assess test statistics (F and *X*^*2*^ for Gaussian GLMs and LMMs, and Deviance (Dev) for binomial GLMs). Estimates and standard error were extracted before dropping the term from the model (Tables S2–S8), and model predictions and credible intervals of figures were simulated with 2000 iterations (*sim* function of the *arm* package version 1.14–4; Gelman et al. 2024).

## Results

### Variation in chronotype

Using the repeated measurements of activity onsets across three years, we found that females left the nest-box at 06:13 ± 0:30 h (mean ± SD) in 2020, 06:06 ± 0:31 h in 2021, and 06:30 ± 0:26 h in 2022. This corresponded to a mean onset of 23 min after sunrise, ranging from 1:49 h (109 min) before to 2:25 h (145 min) after sunrise. The standardised onset (i.e. relative to conspecifics on the same date) deviated from the mean by −4.77 to + 4.00 standard deviation units. These onsets were significantly repeatable within (*r* = 0.210; *Female_Year *_*n*=*220*_: *Χ*^2^_1,*n*=2316_ = 9.654, *p* = 0.002) and across years (*r* = 0.144; *Female*_*n*=*184*_: *Χ*^2^_1,*n*=2316_ = 10.444, *p* = 0.001). The remaining within-individual variation was neither explained by the date of measurement (interaction *Year:April day*: *Χ*^2^_2,*n*=2316_ = 0.494, *p* = 0.781), *April day* (*Χ*^2^_1,*n*=2316_ = 0.184, *p* = 0.668), *Year* (*Χ*^2^_2,*n*=2316_ = 3.008, *p* = 0.222), nor the nest-box used (*Nest-box *_*n*=*162*_: *r* = 0.000, *Χ*^2^_1,*n*=2316_ = 0.000, *p* = 1.000; Table S2). The zero-variance explained by *Nest-box* was likely confounded by the correlation with *Female* (73% nest-boxes occupied by the same female) and with *Female_Year* (69% nest-boxes occupied by one female in one year only). In contrast, *Breeding day* (relative to hatch date) had a quadratic relationship with the standardised onsets (*Breeding day*^*2*^: *Χ*^2^_1,*n*=2316_ = 54.533, *p* < 0.001; *Breeding day*: *Χ*^2^_1,*n*=2316_ = 38.943, *p* < 0.001) so that the onset of activity became later closer to hatching date and earlier again after hatching. The mean chronotypes of each female (which we used for the relationship with fitness) were normally distributed, ranging from −1.20 (earliest) to + 1.16 standard deviation units (latest chronotype) compared to the conspecifics.

### Fitness parameters

**Fig. 1:**
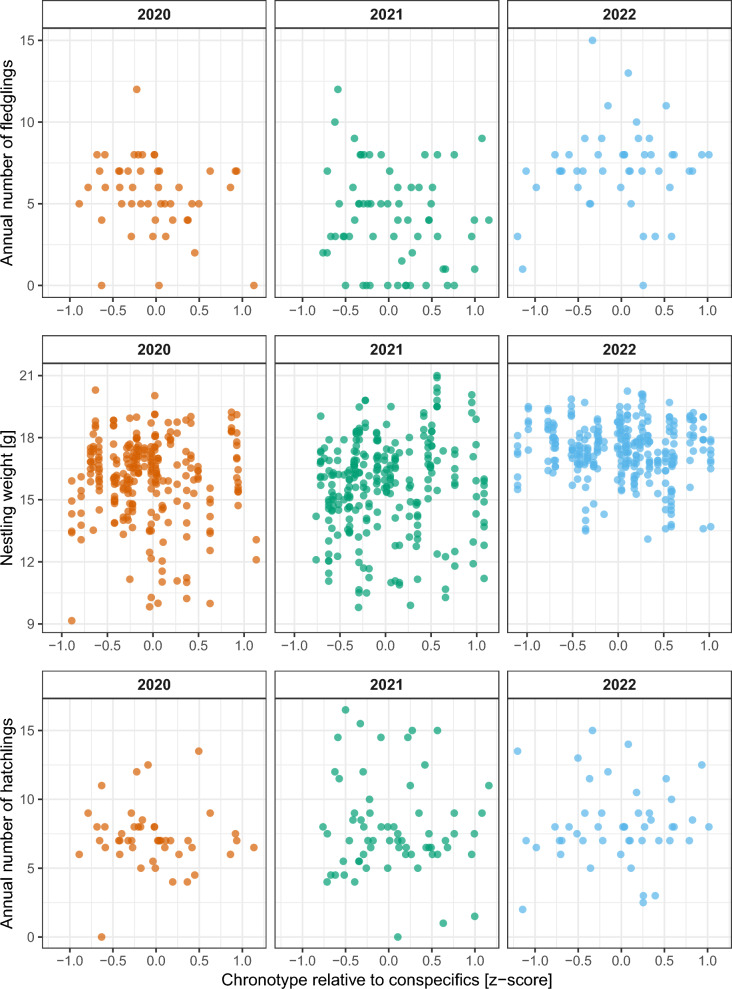
Reproductive outcomes in relation to maternal chronotype. Shown are the number of fledglings (top), body weight of individual nestlings (middle), and the number of hatchlings (bottom). The number of hatchlings were assessed using count + devation/2 (see Fitness and life-history parameters). Chronotypes for each female great tit are standardised mean onsets of activity relative to conspecifics (*z* score)

Fledge success and the number of fledglings were not significantly related to chronotype (fledge success: Dev_1,*n*=157_ =  − 2.233, *p* = 0.135; number of fledglings: − 0.373 ± 0.424, F_1,158_ = 0.775, *p* = 0.380) and its quadratic equivalent in any of the three years (Fig. 1 top, Table S3). Both traits were significantly different between years (fledge success: Dev_2,*n*=157_ =  − 8.205, *p* = 0.017; number of fledglings: F_2,157_ = 17.013, *p* < 0.001), and a relatively late lay date increased the probability of successful fledging per year (Dev_1,*n*=157_ =  − 6.811, *p* = 0.009) but not the number of fledglings (F_1,158_ = 0.473, *p* = 0.493).

Similarly, none of the nestlings’ biometrics were significantly related to female chronotype (weight: −0.025 ± 0.247 g, *Χ*^2^_1,*n*=845_ = 0.013, *p* = 0.911, Fig. 1 middle; tarsus: -1.517 ± 1.203 mm, *Χ*^2^_1,*n*=682_ = 1.682, *p* = 0.195; P3 feather: − 4.362 ± 6.019 mm, *Χ*^2^_1,*n*=835_ = 0.556, *p* = 0.456) and its quadratic equivalent in any of the three years (Table S4). Nestling condition differed between years (weight: *Χ*^2^_2,*n*=845_ = 18.595, *p* < 0.001; tarsus: *Χ*^2^_2,*n*=682_ = 10.312, *p* = 0.006; P3 feather: *Χ*^2^_2,*n*=835_ = 12.389, *p* = 0.002) and was higher (i.e. heavier and larger) at a later chick day and with more chicks fledged per female (Table S4). Nestling weight had a non-significant quadratic relationship with *Time of day* (*Time*^*2*^: 0.031 ± 0.018 g, *Χ*^2^_1,*n*=845_ = 3.021, *p* = 0.082; *Time*: 0.057 ± 0.050 g, *Χ*^2^_1,*n*=845_ = 1.375, *p* = 0.241), likely due to a lack of very early morning measurements (earliest 10:14) when weights are expected to be the lowest (Macleod et al. 2005). Weight was unrelated to the centred hatch date of the brood (0.005 ± 0.012 g, *Χ*^2^_1,*n*=845_ = 0.189, *p* = 0.664) but tarsus and P3 feather length were significantly larger when the brood hatched relatively later (tarsus: 0.183 ± 0.052 mm, *Χ*^2^_2,*n*=682_ = 12.114, *p* = 0.001, P3 feather: 0.599 ± 0.294 mm, *Χ*^2^_2,*n*=835_ = 4.295, *p* = 0.038). There were no significant differences in the number of hatchlings per year between the chronotypes (− 0.158 ± 0.433, F_1,158_ = 0.133, *p* = 0.716), years (F_2,157_ = 1.305, *p* = 0.274), their interaction, or any quadratic equivalents (Fig. 1 bottom, for details see Table S3). The number of hatchlings was, however, higher with a relatively earlier lay date (− 0.140 ± 0.030, F_1,158_ = 22.002, *p* < 0.001). There was no difference in the findings when excluding the two unsuccessful females.

Female body weight during chick provisioning was not significantly related to chronotype (0.111 ± 0.107 g, F_1,152_ = 1.078, *p* = 0.301) and its quadratic equivalent (Table S5), though this potentially differed between years (*Chronotype:Year*: F_2,151_ = 2.478, *p* = 0.088). Later females had non-significantly higher body weight in the challenging spring of 2021 but not in the other two years (*posthoc* not shown). Additionally, we found that body weight non-significantly increased with *Time of day* (F_1,152_ = 3.637, *p* = 0.058). Females with a larger tarsus were 0.049 ± 0.010 g heavier (F_1,152_ = 26.358, *p* < 0.001), and weights differed between years (F_2,151_ = 4.991, *p* = 0.008).

### Life-history traits

**Fig. 2:**
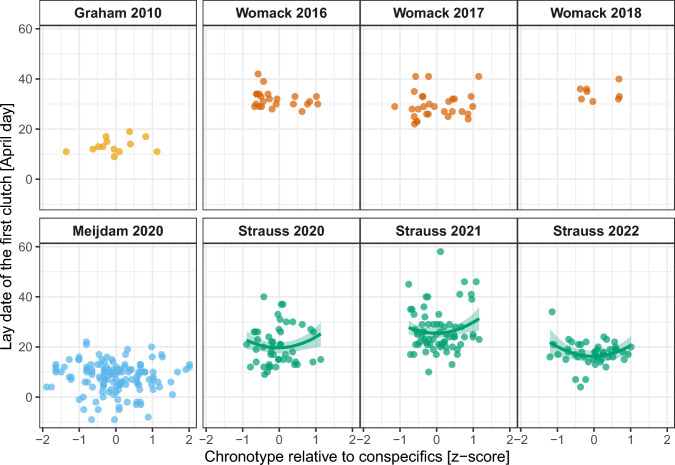
Lay date of the first clutch in relation to maternal chronotype for data from the present study and re-analysed data from earlier studies. Chronotypes for each female great tit are standardised mean onsets of activity relative to conspecifics (*z* score). Lines and shades represent model predictions and 95% credible intervals. Colours and panels represent different studies and years of data collection (Graham et al. 2017a; Meijdam et al. 2022c; Womack et al. 2023b)

We found a significant effect of quadratic chronotype on the lay date of the first clutch (*Chronotype*^*2*^: 4.095 ± 0.821 days, F_1,163_ = 5.058, *p* = 0.026; *Chronotype*: 0.239 ± 1.146 days, F_1,163_ = 0.044, *p* = 0.835; see Fig. 2, Table S6), whereby extremely early and extremely late chronotypes laid their first egg later in the season. While the interaction with year was not significant (*Chronotype*^*2*^*:Year*: F_2,162_ = 2.376, *p* = 0.096), the quadratic relationship might be most strongly in the challenging spring of 2021 (*posthoc* not shown). Lay date significantly differed between years (F_2,162_ = 23.059, *p* < 0.001). In contrast, chronotype was not significantly related to the size of the first clutch (0.091 ± 0.223, F_1,163_ = 0.166, *p* = 0.684) and the probability to initiate a second brood (Dev_1,*n*=131_ =  − 0.533, *p* = 0.465). This was also true for their interactions with year and any quadratic equivalents (Table S6). There was no year effect on clutch size (F_2,162_ = 0.428, *p* = 0.653) but a non-significant difference for the probability of a second brood (Dev_2,*n*=131_ = −5.568, *p* = 0.062) with lower probability in 2020. Further, a later lay date of the first clutch decreased clutch size (− 0.055 ± 0.015, F_1,163_ = 13.184, *p* < 0.001) as well as the probability of a second brood (Dev_1,*n*=131_ =  − 9.128, *p* = 0.003).

### Re-analyses of other datasets using standardised chronotype

For the comparison of our results to other studies, we re-analysed three datasets using our measure of chronotype (described above). There was no significant relationship between female chronotype and the number of fledglings in Womack et al.’s dataset (2023a; −0.722 ± 0.543, F_1,59_ = 1.768, *p* = 0.189) when using the activity onset relative to conspecifics (Table S7) compared to the original analysis using onsets relative to sunrise. With regard to lay date, we could not find a relationship with chronotype in any of the datasets (Womack: −0.098 ± 0.980, F_1,59_ = 0.010, *p* = 0.920; Graham: 1.151 ± 1.320, F_1,12_ = 0.760, *p* = 0.402; Meijdam: −0.341 ± 0.642, F_1,137_ = 0.282, *p* = 0.597; Tables S7–8 and Fig. 2) in contrast to some of the original results (Graham et al. 2017b; egg-laying subset of Meijdam et al. 2022b).

## Discussion

We tested whether female great tits’ chronotypes were related to fitness and life-history traits. Our measure of chronotype, the standardised onset of daily activity, was moderately but significantly repeatable within and across years, and independent from variation between dates of measurement and years. Female chronotype was not significantly related to fitness, nestling biometrics, or female body condition, and neither to most of the life-history traits. Only lay date of the first clutch showed a significant quadratic relationship, where extremely early or late chronotype females laid their first egg later in the season. Thus, our results do not show clear evidence for direct fitness consequences of female chronotype.

### No effect of chronotype on overall annual fitness

We studied a number of fitness components and found no effect of female chronotype on the ability to fledge at least one chick per year (i.e. fledge success), which is in accordance with previous findings in the same species (Womack et al. 2023a). We also did not find an effect of chronotype on the number of fledglings, similar to a study on female starlings (Maury et al. 2020), but contrary to Womack et al. (2023a), where early-active female great tits had more fledglings. Apart from potential differences between populations, Womack et al. (2023a) defined chronotype as activity onset relative to sunrise and accounted for seasonal effects by including hatch date in their statistical analyses. However, when re-analysing their dataset using our standardised onsets and statistical models, the chronotype effect on the number of fledglings was no longer present. A recent experiment demonstrated that data handling and analyses practices can have an extreme impact on the effect size and results even when using the same data (Gould et al. 2025). Here, the discrepancy to the original analysis of Womack et al. (2023a) likely comes from those differences in data processing and statistical modelling: First, the standardisation of chronotype in our study accounted differently for within-individual effects of seasonality and day-to-day variation, and therefore increased the between-individual variation in chronotype (see below). Thereby, individuals classified as extreme phenotypes using onset relative to sunrise might become assigned less extreme chronotypes during our standardisation because they were only compared to individuals experiencing the same date-specific conditions. Second, in our re-analysis, we included year-centred lay date, which accounted for between-year differences in reproductive timing, and found a significant decrease in the number of fledglings with later lay date. In contrast, Womack et al.’s model used a linear and quadratic term of hatch date instead and found no seasonal effect on the number of fledglings (2023a). Altogether, our standardisation approach does not support an effect of female chronotype on fitness in both great tit populations.

### No compensatory effects or differences in investment across breeding stages

The lack of a relationship between chronotype and the fledging number could result from balancing selection or different investment across breeding stages and parental care. However, there were no significant differences in the investment into current reproduction and its breeding stages for our chronotype data: First, we did not find chronotype effects on the size of the first clutch or the initiation of a second brood. Similar results were found for clutch sizes in other songbirds (Murphy et al. 2008; Steinmeyer et al. 2013; Maury et al. 2020), while early-active female starlings were more likely to initiate a second brood (Maury et al. 2020). Second, being earlier or later was not significantly related to the number of hatchlings. Although incubating females trade off foraging for self-maintenance against warming of the eggs (Reid et al. 2000; Vedder 2012), chronotype might not be as important for hatch success as other incubation parameters such as nest attentiveness. For example, extended off-bouts, due to harsh weather conditions, can decrease hatching rates (MacDonald et al. 2013). Our females might have avoided the rapid cooling of the eggs by shortening the first off-bout in the early morning, balancing nest attentiveness and foraging demands, or altered incubation patterns when fed by their partner (Amininasab et al. 2016; Nilsson and Smith 1988, but see Matysioková and Remeš, 2010) or to manipulate hatch date (Vedder 2012; Simmonds et al. 2017). Third, nestlings’ developmental condition, measured as tarsus and P3 feather length as well as body mass (Orell 1983; Verboven and Visser 1998; Radersma et al. 2011), was not significantly related to female chronotype here. Likewise, nestling weight was independent of their mother’s chronotype in other studies (Pagani-Núñez and Senar 2016; Maury et al. 2020; Womack et al. 2023a). This could be due to the low importance of the time of day for provisioning rates compared to other environmental and intrinsic brood factors such as brood size and chick age (Barba et al. 2009). Alternatively, females could have prioritised future reproduction and survival by investing in self-maintenance. Here, we found no strong link between chronotype and female condition. Body weight of small songbirds fluctuates substantially within short-term periods (Haftorn 1989) because of strategic responses to environmental conditions and their predictability (Bednekoff et al. 1994; Krams 2000). This might explain why our data showed a trend of late females to be heavier but only in the rainy and cold spring of 2021. Yet, a measurement of long-term condition, the yellow feather colouration, was also unrelated to the activity onset during provisioning in females (Pagani-Núñez and Senar 2016). Overall, chronotype might not be a crucial strategy for females to alter their investment and outcome across different breeding stages.

### Standardisation captures day-to-day variation in the environment but not in breeding stage

A possibility for the lack of fitness consequences in our analyses could come from the rather large, unexplained variation in the standardised onsets used for chronotype. We standardised the onset of activity in relation to conspecifics on the same day to account for within- and between-individual variation from seasonal and other environmental effects. Although this reduced within-individual variation, the adjusted repeatability was still moderate with *r* = 0.21 within and *r* = 0.14 across years. This magnitude is still reasonable for a behavioural trait (Bell et al. 2009) and falls within the range of other great tit studies ranging from *r* = 0.13 in winter (Stuber et al. 2015) over 0.27–0.54 during egg laying (Meijdam et al. 2022a, b) to 0.32 during incubation (Womack et al. 2023a). On the other hand, our standardised onsets were still dependent on the day of breeding, where onsets got later towards hatching and advanced again thereafter, a pattern previously observed in tits (Schlicht and Kempenaers 2020; Womack et al. 2023a; Strauß et al. 2024). This intrinsic effect of breeding stage could be accounted for by the inclusion of a seasonal component (e.g. lay date or hatch date) into the standardisation process resulting in the comparison of individuals at the same breeding day, as demonstrated by Schlicht et al. (2023). However, this method requires large amounts of data and synchronised breeding of the females to avoid a strong reduction of data available for standardisation. Alternatively, raw onsets could be modelled together with the fitness component in bivariate models; then their covariation could reveal potential correlations (Houslay and Wilson 2017). These statistical analyses, however, are often challenging and complex to model and interpret because different model structures and error distributions need to be combined. A current paper using this method still failed to find fitness consequences for female chronotypes in great tits (Meijdam et al. 2025). Our approach focussed on the reduction of environmental confounders while retaining the large sample size and was able to reduce the associated within-individual variation and to increase the consistency of the chronotype trait. Nevertheless, we call for further development to improve the extraction of individuals’ true chronotypes.

### A quadratic relationship with lay date could benefit intermediate chronotypes in early springs

Surprisingly, we found a quadratic relationship between lay date and chronotype, in which extremely early and late chronotypes laid their first egg later in the season. While there was no significant difference between years, this relationship might have been driven by the data collected in the wet spring of 2021. Other studies found either earlier lay dates with earlier chronotypes (Murphy et al. 2008; Graham et al. 2017b) or no relationship (Steinmeyer et al. 2013; Maury et al. 2020). These studies used onsets relative to sunrise that could confound relationships with breeding timing due to the comparison of individuals measured at different timepoints (Meijdam et al. 2022b). Re-analysing three publicly available datasets (Graham et al. 2017a; Meijdam et al. 2022c; Womack et al. 2023b) using our standardised chronotype approach, no positive association between time of breeding and chronotype remained significant, and also no quadratic relationship was found. These inconclusive findings on the quadratic effect might be due to the leftover within-individual variation in the standardised onsets (see above), the different statistical models used (Gould et al. 2025), or even differences in biology and ecological conditions between populations.

Seasonal and daily timing could be linked via the internal circadian clock and its genetic background, where the endogenous rhythm for annual timing adjusts to daylength (Helm 2021). But the mechanism is not fully understood yet: while individuals differ in their clock period length, it is unclear how much is actually reflected in chronotypes in the wild (Tomotani et al. 2023 versus 2024), and a link to lay date has also not been found yet (Helm and Visser 2010). If there is a phenotypic and genetic correlation between lay date and chronotype, chronotype could affect fitness indirectly via the selection on lay date. In our study, a later lay date significantly increased fledge success but decreased clutch size, the number of hatchlings, and the probability to initiate a second brood. The number of fledglings was not significantly affected by lay date. While these results seem inconclusive, the selection of lay date in this study population is known to fluctuate across years; it had been overall balanced until 2000, after which earlier breeding got increasingly beneficial (Jantzen and Visser 2023). Thus, indirect effects through lay date could have benefitted the intermediate chronotypes through selection of early breeders in the past 20 years. As early breeding is becoming more beneficial due to climate change (Visser et al. 2012), a selection for intermediate chronotypes could potentially decrease between-individual variation in the future.

## Conclusion

In conclusion, female chronotype, standardised within date, was not significantly related to breeding success and most life-history traits. The method for chronotype extraction, however, plays a key role in subsequent analyses. Caution must be taken to disentangle diel timing from its variation over the breeding season. While we were able to remove some of the within-individual variation in our data, the individuals’ true chronotype might still differ or might simply have low consistency. Overall, it is possible that chronotype is no longer adaptive but persists through a lack of selection against chronotype or a link to other traits under selection (Jabbur et al. 2024). In fact, we found that extreme chronotypes breed later in the season in our study population, and variation could therefore be maintained as a by-product of the lay-date selection. Indirect effects of chronotype via lay date could then favour intermediate chronotypes in view of an advancing spring. Further studies are needed to reveal the mechanistic link between annual and diel timing through the internal clock. Moreover, we limited our analysis to one breeding season per female, though great tits can breed over multiple years. If selection fluctuates temporally and spatially, different chronotypes could have similar lifetime reproductive success, maintaining variation in chronotypes as suggested for other phenotypic traits (Boyce and Perrins 1987; Jantzen and Visser 2023). Selection pressures might differ between breeding seasons or at other times of the year such as overwintering affecting survival. Future research might investigate survival rates at other times of the year and potential effects of fluctuating selection on a longer timescale.

## Supplementary Information

Below is the link to the electronic supplementary material.

Supplementary file1 (DOCX 442 KB)

## Data Availability

Data and statistic script will be made available here: 10.6084/m9.figshare.28966850. Additional raw data are available here: 10.6084/m9.figshare.24973095.

## Code availability

Codes for the nest temperature processing are available in the Supporting Information 2 of Strauß et al. 2024: 10.1002/jez.2787. For statistic R code, see above.

## References

[CR1] Álvarez E, Barba E (2014) Within and between population variations of incubation rhythm of great tits *Parus major*. Behaviour 151:1827–1845. 10.1163/1568539X-00003218

[CR2] Amininasab SM, Kingma SA, Birker M et al (2016) The effect of ambient temperature, habitat quality and individual age on incubation behaviour and incubation feeding in a socially monogamous songbird. Behav Ecol Sociobiol 70:1591–1600. 10.1007/S00265-016-2167-2 27546954 10.1007/s00265-016-2167-2 PMC4977336

[CR92] Anderson SR, Wiens JJ (2017) Out of the dark: 350 million years of conservatism and evolution in diel activity patterns in vertebrates. Evolution 71(8):1944–1959. 10.1111/evo.13284 28636789 10.1111/evo.13284

[CR3] Barba E, Atiénzar F, Marín M et al (2009) Patterns of nestling provisioning by a single-prey loader bird, great tit *Parus major*. Bird Study 56:187–197. 10.1080/00063650902792049

[CR4] Bates D, Mächler M, Bolker B, Walker S (2015) Fitting linear mixed-effects models using lme4. J Stat Softw 67:1–48. 10.18637/jss.v067.i01

[CR5] Bednekoff PA, Biebach H, Krebs J (1994) Great tit fat reserves under unpredictable temperatures. J Avian Biol 25:156–160

[CR6] Bell AM, Hankison SJ, Laskowski KL, Bell AM (2009) The repeatability of behaviour: a meta-analysis. Anim Behav 77:771–783. 10.1016/j.anbehav.2008.12.022 24707058 10.1016/j.anbehav.2008.12.022 PMC3972767

[CR7] Bermúdez-Cuamatzin E, Delamore Z, Verbeek L et al (2020) Variation in diurnal patterns of singing activity between urban and rural great tits. Front Ecol Evol 8:1–15. 10.3389/fevo.2020.00246

[CR8] Bonnot NC, Couriot O, Berger A et al (2020) Fear of the dark? Contrasting impacts of humans versus lynx on diel activity of roe deer across Europe. J Anim Ecol 89:132–145. 10.1111/1365-2656.13161 31799691 10.1111/1365-2656.13161

[CR9] Boulton RL, Cassey P (2012) How avian incubation behaviour influences egg surface temperatures: relationships with egg position, development and clutch size. J Avian Biol 43:289–296. 10.1111/J.1600-048X.2012.05657.X

[CR10] Boyce MS, Perrins CM (1987) Optimizing great tit clutch size in a fluctuating environment. Ecology 68:142–153. 10.2307/1938814

[CR11] Capilla-Lasheras P (2018) Incr: a new R package to analyse incubation behaviour. J Avian Biol 49:e01710. 10.1111/jav.01710

[CR12] Coe BH, Beck ML, Chin SY et al (2015) Local variation in weather conditions influences incubation behavior and temperature in a passerine bird. J Avian Biol 46:385–394. 10.1111/jav.00581

[CR13] Cooper CB, Hochachka WM, Butcher G, Dhondt AA (2005) Seasonal and latitudinal trends in clutch size: thermal constraints during laying and incubation. Ecology 86:2018–2031. 10.1890/03-8028

[CR14] Diez-Méndez D, Sanz JJ, Barba E (2021) Impacts of ambient temperature and clutch size on incubation behaviour onset in a female-only incubator songbird. Ibis 163:1056–1071. 10.1111/ibi.12937

[CR15] Dominoni DM, Helm B, Lehmann M et al (2013) Clocks for the city: circadian differences between forest and city songbirds. Proc R Soc Lond B Biol Sci 280:20130593. 10.1098/rspb.2013.0593 10.1098/rspb.2013.0593 PMC3774226 23740778

[CR16] Duranton C, Gaunet F (2016) Behavioural synchronization from an ethological perspective: overview of its adaptive value. Adapt Behav 24:181–191. 10.1177/1059712316644966

[CR17] Engert ER, Andreasson F, Nord A, Nilsson JÅ (2024) Using metabolic data to investigate the role of brood size in the development of endothermy. J Avian Biol. 10.1111/JAV.03301

[CR18] Gelman A, Su Y-S, Yajima M, Hill J, Pittau MG, Kerman J, Zheng T, Dorie V (2024) arm: Data Analysis Using Regression and Multilevel/Hierarchical Models (1.14-4) [R package]. CRAN. 10.32614/CRAN.package.arm

[CR19] Gould E, Fraser HS, Parker TH et al (2025) Same data, different analysts: variation in effect sizes due to analytical decisions in ecology and evolutionary biology. BMC Biol 23:35. 10.1186/s12915-024-02101-x 39915771 10.1186/s12915-024-02101-x PMC11804095

[CR20] Graham JL, Cook NJ, Hau M, Greives TJ (2017a) Data from: early to rise, early to breed: a role for daily rhythms in seasonal reproduction. Dyrad. 10.5061/dryad.h297n

[CR21] Graham JL, Cook NJ, Needham KB et al (2017b) Early to rise, early to breed: a role for daily rhythms in seasonal reproduction. Behav Ecol 28:1266–1271. 10.1093/beheco/arx088

[CR22] Greives TJ, Kingma SA, Kranstauber B et al (2015) Costs of sleeping in: circadian rhythms influence cuckoldry risk in a songbird. Funct Ecol 29:1300–1307. 10.1111/1365-2435.12440

[CR23] Haftorn S (1988) Incubating female passerines do not let the egg temperature fall below the “physiological zero temperature” during their absences from the nest. Ornis Scand 19:97–110

[CR24] Haftorn S (1989) Seasonal and Diurnal Body Weight Variations in Titmice, Based on Analyses of Individual Birds. Wilson Bull 101:217–235

[CR25] Hau M, Dominoni DM, Casagrande S et al (2017) Timing as a sexually selected trait: the right mate at the right moment. Philos Trans R Soc Lond B Biol Sci 372:20160249. 10.1098/rstb.2016.0249 28993493 10.1098/rstb.2016.0249 PMC5647276

[CR26] Helm B (2021) Clocks and Calendars in Birds. In: Ebling FJP, Piggins HD (eds) Neuroendocrine Clocks and Calendars. Masterclass in Neuroendocrinology. Springer Cham, Cham, pp 119–142

[CR27] Helm B, Visser ME (2010) Heritable circadian period length in a wild bird population. Proc R Soc Lond B Biol Sci 277:3335–3342. 10.1098/rspb.2010.0871 10.1098/rspb.2010.0871 PMC2981934 20534621

[CR28] Houslay TM, Wilson AJ (2017) Avoiding the misuse of BLUP in behavioral ecology. Behav Ecol 28(4):948–952. 10.1093/beheco/arx023 29622923 10.1093/beheco/arx023 PMC5873244

[CR29] Jabbur ML, Dani C, Spoelstra K et al (2024) Evaluating the adaptive fitness of circadian clocks and their evolution. J Biol Rhythms 39(2):1–20. 10.1177/07487304231219206 10.1177/07487304231219206 PMC10994774 38185853

[CR30] Jantzen CC, Visser ME (2023) Climate change does not equally affect temporal patterns of natural selection on reproductive timing across populations in two songbird species. Proc R Soc Lond B Biol Sci 290:20231474. 10.1098/rspb.2023.1474 10.1098/rspb.2023.1474 PMC10581764 37848060

[CR31] Kacelnik A (1979) The foraging efficiency of great tits (*Parus major* L.) in relation to light intensity. Anim Behav 27:237–241

[CR32] Kluyver HN (1970) Regulation of numbers in populations of Great Tits (Parus m. major). In: Proceedings of the Advanced Study Institute on “Dynamics of Numbers in Populations.” pp 507–523

[CR33] Kontogiannis JE (1967) Day and night changes in body weight of the white-throated sparrow, *Zonotrichia albicollis*. Auk 84:390–395. 10.2307/4083088

[CR34] Krams I (2000) Length of feeding day and body weight of great tits in a single- and two-predator environment. Behav Ecol Sociobiol 48:147–153. 10.1007/S002650000214/METRICS

[CR35] Laine VN, Atema E, Vlaming P et al (2019) The genomics of circadian timing in a wild bird, the Great Tit (*Parus major*). Front Ecol Evol 7:152. 10.3389/fevo.2019.00152

[CR36] Le Clercq L-S, Bazzi G, Cecere JG et al (2023) Time trees and clock genes: a systematic review and comparative analysis of contemporary avian migration genetics. Biol Rev 98:1051–1080. 10.1111/brv.12943 36879518 10.1111/brv.12943

[CR37] Lemaître JF, Berger V, Bonenfant C et al (2015) Early-late life trade-offs and the evolution of ageing in the wild. Proc R Soc Lond B Biol Sci 282:20150209. 10.1098/RSPB.2015.0209 10.1098/rspb.2015.0209 PMC4426628 25833848

[CR38] MacDonald EC, Camfield AF, Jankowski JE, Martin K (2013) Extended incubation recesses by alpine-breeding Horned Larks: a strategy for dealing with inclement weather? J Field Ornithol 84:58–68. 10.1111/JOFO.12006

[CR39] Macleod R, Gosler AG, Cresswell W (2005) Diurnal mass gain strategies and perceived predation risk in the great tit *Parus major*. J Anim Ecol 74:956–964. 10.1111/J.1365-2656.2005.00993.X

[CR40] Martin TE (2002) A new view of avian life-history evolution tested on an incubation paradox. Proc R Soc Lond B Biol Sci 269:309–316. 10.1098/rspb.2001.1879 10.1098/rspb.2001.1879 PMC1690888 11839200

[CR41] Martorell-Barceló M, Campos-Candela A, Alós J (2018) Fitness consequences of fish circadian behavioural variation in exploited marine environments. PeerJ 6:e4818. 10.7717/peerj.4814 29796349 10.7717/peerj.4814 PMC5961624

[CR42] Matysioková B, Remeš V (2010) Incubation feeding and nest attentiveness in a socially monogamous songbird: role of feather colouration, territory quality and ambient environment. Ethology 116:596–607. 10.1111/J.1439-0310.2010.01776.X

[CR43] Maury C, Serota MW, Williams TD (2020) Plasticity in diurnal activity and temporal phenotype during parental care in European starlings, *Sturnus vulgaris*. Anim Behav 159:37–45. 10.1016/j.anbehav.2019.11.004

[CR44] McNamara JM, Houston AI, Lima SL (1994) Foraging routines of small birds in winter: a theoretical investigation. J Avian Biol 25:287. 10.2307/3677276

[CR45] Meijdam M, Müller W, Eens M (2022a) Intrinsic individual variation in daily activity onset and plastic responses on temporal but not spatial scales in female great tits. Sci Rep 12:18022. 10.1038/s41598-022-22935-1 36289438 10.1038/s41598-022-22935-1 PMC9605954

[CR46] Meijdam M, Müller W, Thys B, Eens M (2022b) No relationship between chronotype and timing of breeding when variation in daily activity patterns across the breeding season is taken into account. Ecol Evol 12:1–11. 10.1002/ece3.9353 10.1002/ece3.9353 PMC9490139 36188525

[CR47] Meijdam M, Müller W, Thys B, Eens M (2022c) Data from: no relationship between chronotype and timing of breeding when variation in daily activity patterns across the breeding season is taken into account. Dyrad. 10.5061/dryad.2rbnzs7rk 10.1002/ece3.9353 PMC9490139 36188525

[CR48] Meijdam M, Eens M, Müller W (2025) Female chronotype is not related to annual and lifetime reproductive success in a free-living songbird. R Soc Open Sci. 10.1098/rsos.250380 40904987 10.1098/rsos.250380 PMC12404840

[CR49] Mertens JAL (1977) Thermal conditions for successful breeding in great tits (*Parus major* L.) I. relation of growth and development of temperature regulation in nestling great tits. Oecologia 28:1–29 28309686 10.1007/BF00346834

[CR50] Murphy MT, Sexton K, Dolan AC, Redmond LJ (2008) Dawn song of the eastern kingbird: an honest signal of male quality? Anim Behav 75:1075–1084. 10.1016/J.ANBEHAV.2007.08.020

[CR51] Nakagawa S, Schielzeth H (2010) Repeatability for Gaussian and non-Gaussian data: a practical guide for biologists. Biol Rev 85:935–956. 10.1111/J.1469-185X.2010.00141.X 20569253 10.1111/j.1469-185X.2010.00141.x

[CR52] Nilsson J-Å, Smith HG (1988) Incubation feeding as a male tactic for early hatching. Anim Behav 36:641–647

[CR53] Norberg RA (1977) An ecological theory on foraging time and energetics and choice of optimal food-searching method. J Anim Ecol 46:511–529. 10.2307/3827

[CR54] Nord A, Cooper CB (2020) Night conditions affect morning incubation behaviour differently across a latitudinal gradient. Ibis 162:827–835. 10.1111/ibi.12804

[CR55] Orell M (1983) Nestling growth in the Great Tit *Parus major* and the Willow Tit *P. montanus*. Ornis Fenn 60:65–85

[CR56] Pagani-Núñez E, Senar JC (2013) One hour of sampling is enough: great tit *Parus major* parents feed their nestlings consistently across time. Acta Ornithol 48:194–200. 10.3161/000164513X678847

[CR57] Pagani-Núñez E, Senar JC (2016) More ornamented great tit *Parus major* fathers start feeding their offspring earlier. Ardea 104:167–176. 10.5253/arde.v104i2.a1

[CR58] Patterson JEH, Ruckstuhl KE (2013) Parasite infection and host group size: a meta-analytical review. Parasitology 140:803–813. 10.1017/S0031182012002259 23425516 10.1017/S0031182012002259 PMC3638372

[CR59] Perrins CM, McCleery RH (1989) Laying dates and clutch size in the Great Tit. Wilson Bull 101:236–253

[CR60] Poesel A, Kunc HP, Foerster K et al (2006) Early birds are sexy: male age, dawn song and extrapair paternity in blue tits, *Cyanistes* (formerly *Parus*) *caeruleus*. Anim Behav 72:531–538. 10.1016/J.ANBEHAV.2005.10.022

[CR61] R Core Team (2024) R: A Language and Environment for Statistical Computing. R Foundation for Statistical Computing. Vienna, Austria. https://www.R-project.org/

[CR62] Radersma R, Tinbergen JM, Komdeur J (2011) Do brood sex ratio, nestling development and sex affect fledging timing and order? An experimental study on great tits. Anim Behav 81:69–75. 10.1016/J.ANBEHAV.2010.09.007

[CR63] Réale D, Garant D, Humphries MM et al (2010) Personality and the emergence of the pace-of-life syndrome concept at the population level. Philos Trans R Soc Lond B Biol Sci 365:4051–4063. 10.1098/rstb.2010.0208 21078657 10.1098/rstb.2010.0208 PMC2992747

[CR64] Reid JM, Monaghan P, Ruxton GD (2000) Resource allocation between reproductive phases: the importance of thermal conditions in determining the cost of incubation. Proc R Soc Lond B Biol Sci 267:37–41. 10.1098/rspb.2000.0963 10.1098/rspb.2000.0963 PMC1690493 10670950

[CR65] Royal Netherlands Meterological Institute (KNMI) (2023) Daggegevens van het weer in Nederland. https://www.knmi.nl/nederland-nu/klimatologie/daggegevens. Accessed 30 Jun 2023

[CR66] Salmela MJ, Weinig C (2019) The fitness benefits of genetic variation in circadian clock regulation. Curr Opin Plant Biol 49:86–93. 10.1016/j.pbi.2019.06.003 31302588 10.1016/j.pbi.2019.06.003

[CR67] Schlicht L, Kempenaers B (2020) The effects of season, sex, age and weather on population-level variation in the timing of activity in Eurasian blue tits *Cyanistes caeruleus*. Ibis 162:1146–1162. 10.1111/ibi.12818

[CR68] Schlicht L, Valcu M, Loës P et al (2014) No relationship between female emergence time from the roosting place and extrapair paternity. Behav Ecol 25:650–659. 10.1093/beheco/aru035

[CR69] Schlicht L, Santema P, Kempenaers B (2023) Start and end of daily activity predict extrapair siring success independently of age in male blue tits. Anim Behav 198:21–31. 10.1016/J.ANBEHAV.2023.01.016

[CR70] Schwartz WJ, Helm B, Gerkema MP (2017) Wild clocks: preface and glossary. Philos Trans R Soc Lond B Biol Sci 372:20170211. 10.1098/RSTB.2017.0211 28993501 10.1098/rstb.2017.0211 PMC5647284

[CR71] Simmonds EG, Sheldon BC, Coulson T, Cole EF (2017) Incubation behavior adjustments, driven by ambient temperature variation, improve synchrony between hatch dates and caterpillar peak in a wild bird population. Ecol Evol 7:9415–9425. 10.1002/ece3.3446 29187978 10.1002/ece3.3446 PMC5696398

[CR72] Smith H, Källander H, Nilsson J (1987) Effect of experimentally altered brood size on frequency and timing of second clutches in the great tit. Auk. 10.1093/auk/104.4.700

[CR73] Stearns SC (1989) Trade-offs in life-history evolution. Funct Ecol 3:259–268

[CR74] Steinmeyer C, Kempenaers B, Mueller JC (2012) Testing for associations between candidate genes for circadian rhythms and individual variation in sleep behaviour in blue tits. Genetica 140:219–228. 10.1007/s10709-012-9673-6 22922941 10.1007/s10709-012-9673-6

[CR75] Steinmeyer C, Mueller JC, Kempenaers B (2013) Individual variation in sleep behaviour in blue tits *Cyanistes caeruleus*: assortative mating and associations with fitness-related traits. J Avian Biol 44:159–168. 10.1111/J.1600-048X.2012.05750.X

[CR76] Strauß AFT, Bosma L, Visser ME, Helm B (2024) Short-time exposure to light at night affects incubation patterns and correlates with subsequent body weight in great tits (*Parus major*). J Exp Zool A Ecol Integr Physiol 341:364–376. 10.1002/jez.2787 38327263 10.1002/jez.2787

[CR77] Stuber EF, Dingemanse NJ, Kempenaers B, Mueller JC (2015) Sources of intraspecific variation in sleep behaviour of wild great tits. Anim Behav 106:201–221. 10.1016/j.anbehav.2015.05.025

[CR78] Tomotani BM, Timpen F, Spoelstra K (2023) Ingrained city rhythms: flexible activity timing but more persistent circadian pace in urban birds. Proc R Soc Lond B Biol Sci 290:20222605. 10.1098/rspb.2022.2605 10.1098/rspb.2022.2605 PMC10188242 37192668

[CR79] Tomotani BM, Strauß AFT, Kishkinev D et al (2024) Circadian clock period length is not consistently linked to chronotype in a wild songbird. Eur J Neurosci 60:5522–5536. 10.1111/ejn.16535 39256897 10.1111/ejn.16535

[CR80] van der Vinne V, Tachinardi P, Riede SJ et al (2019) Maximising survival by shifting the daily timing of activity. Ecol Lett 22:2097–2102. 10.1111/ele.13404 31617283 10.1111/ele.13404 PMC6899458

[CR81] Vedder O (2012) Individual birds advance offspring hatching in response to increased temperature after the start of laying. Oecologia 170:619–628. 10.1007/s00442-012-2335-7 22569557 10.1007/s00442-012-2335-7

[CR82] Verboven N, Verhulst S (1996) Seasonal variation in the incidence of double broods: the date hypothesis fits better than the quality hypothesis. J Anim Ecol 65:264–273. 10.2307/5873

[CR83] Verboven N, Visser ME (1998) Seasonal variation in local recruitment of Great Tits: the importance of being early. Oikos 81:511–524

[CR84] Verhagen IC, Gienapp P, Laine VN et al (2019) Genetic and phenotypic responses to genomic selection for timing of breeding in a wild songbird. Funct Ecol 33:1708–1721. 10.1111/1365-2435.13360

[CR85] Verhulst S, Tinbergen JM, Daan S (1997) Multiple breeding in the Great Tit. A trade-off between successive reproductive attempts? Funct Ecol 11:714–722. 10.1046/j.1365-2435.1997.00145.x

[CR86] Visser ME, Verboven N (1999) Long-term fitness effects of fledging date in Great Tits. Oikos 85:445–450

[CR87] Visser ME, te Marvelde L, Lof ME (2012) Adaptive phenological mismatches of birds and their food in a warming world. J Ornithol 153:75–84. 10.1007/s10336-011-0770-6

[CR88] Webb DR (1987) Thermal tolerance of avian embryos: a review. Condor 89:874–898

[CR89] West AC, Bechtold DA (2015) The cost of circadian desynchrony: evidence, insights and open questions. BioEssays 37:777–788. 10.1002/bies.201400173 26010005 10.1002/bies.201400173 PMC4973832

[CR90] Womack RJ, Capilla-Lasheras P, Mcglade CLO et al (2023a) Reproductive fitness is associated with female chronotype in a songbird. Anim Behav 205:65–78. 10.1016/j.anbehav.2023.08.018

[CR91] Womack RJ, Capilla-Lasheras P, McGlade CLO et al (2023b) Data from: reproductive fitness is associated with female chronotype in a songbird. Zenodo. 10.5281/ZENODO.7967106

