# Supplementary material for: Female chronotype relates to lay date but not fitness in an island population of great tits
Source: Oecologia. 2026 Jan 10;208(2):22. doi: 10.1007/s00442-025-05857-3 (PMC12789178; doi:10.1007/s00442-025-05857-3)
Supplement: Supplementary file 1 — Supplementary file1 (DOCX 442 KB) [file 442_2025_5857_MOESM1_ESM.docx]

*Oecologia*

Supplementary Information 1

Female chronotype relates to lay date but not fitness in an island population of great tits

Aurelia F. T. Strauß, Barbara M. Tomotani, Barbara Helm and Marcel E. Visser

Corresponding author Aurelia F. T. Strauß: [a.strauss@rug.nl](mailto:a.strauss@rug.nl)

## Table of Content

1. **Activity data processing: Determining on- and off-bouts using changes in nest temperatures**
2. **Full Estimates and Statistics of Repeatability, Fitness Parameters and Life History Traits**

Supplementary tables S1 – S5

1. **Re-analyses of other datasets using standardised chronotype**

Supplementary tables S6 – S7

## References

Capilla-Lasheras P (2018) incR: a new R package to analyse incubation behaviour. J Avian Biol 49:e01710. https://doi.org/10.1111/jav.01710

Diez‐Méndez D, Sanz JJ, Barba E (2021) Impacts of ambient temperature and clutch size on incubation behaviour onset in a female‐only incubator songbird. Ibis 163:1056–1071. https://doi.org/10.1111/ibi.12937

Graham JL, Cook NJ, Hau M, Greives TJ (2017) Data from: Early to rise, early to breed: a role for daily rhythms in seasonal reproduction. Dyrad. https://doi.org/10.5061/dryad.h297n

Kacelnik A (1979) The foraging efficiency of great tits (Parus major L.) in Relation to light intensity. Anim Behav 27:237–241

Kishida Y (1989) Changes in Light Intensity at Twilight and Estimation of the Biological Photoperiod. JARQ 22:247–252

Meijdam M, Müller W, Thys B, Eens M (2022) Data from: No relationship between chronotype and timing of breeding when variation in daily activity patterns across the breeding season is taken into account. Dyrad. https://doi.org/10.5061/dryad.2rbnzs7rk

Podlas K, Richner H (2013) Partial incubation and its function in great tits (Parus major) - an experimental test. Behavioral Ecology 24:643–649. https://doi.org/10.1093/beheco/ars224

R Core Team (2023) R: A Language and Environment for Statistical Computing. R Foundation for Statistical Computing. Vienna, Austria. https://www.R-project.org/

Smith JA, Cooper CB, Reynolds SJ (2015) Advances in techniques to study incubation. In: Nests, Eggs, and Incubation. Oxford University Press, pp 179–195

Strauß AFT, Bosma L, Visser ME, Helm B (2024) Short‐time exposure to light at night affects incubation patterns and correlates with subsequent body weight in great tits (*Parus major*). J Exp Zool A Ecol Integr Physiol 341:364–376. https://doi.org/10.1002/jez.2787

Womack RJ, Capilla-Lasheras P, McGlade CLO, et al (2023) Data from: Reproductive fitness is associated with female chronotype in a songbird. Zenodo. https://doi.org/10.5281/ZENODO.7967106

## 1. Activity data processing: Determining on- and off-bouts using changes in nest temperatures

To determine on- and off-bouts we used the R pipeline in Strauß et al. (2024) that evaluates the changes in nest temperature in relation to thresholds based on temperature variation in the nest and the environment during night and day.

*1.1 Extraction of temperature variation of nest and environment*

In preparation for the comparison of temperature fluctuations within nest and day, we extracted patterns of cooling and warming for ambient and nest temperature, separately, by calculating the difference between consecutive temperature recordings (hereafter called: drop and rise, respectively). For slow and steady cooling and warming of the nest, we additionally assessed the cumulative differences of cooling and warming (i.e. additive differences until sign changed, and hereafter called: cumulative cooling and warming, respectively), and the maximum difference for each of these on- and off-bouts. We also extracted the differences between ambient and nest temperature, and the subsequent differences between consecutive timepoints (i.e. the pattern of the temperature differences).

*1.2 Extraction of nest- and day-specific thresholds*

Then, nest-specific thresholds were extracted from night-time and daytime references of each day to assign off- and on-bouts (see below). In this pipeline here, we defined the night reference phase as from after nautical dusk to before nautical dawn (i.e. the sun 12° below the horizon; hereafter called night-time). The day reference phase, in turn, corresponded the rest of the day, respectively. At night females are expected to roost and partially incubate on the nest (Podlas and Richner 2013), while leaving the nest-box for foraging seems unlikely due to the low light levels (Kacelnik 1979; Kishida 1989). Some nights had particularly low nest temperatures (i.e. with minimum temperatures below 15 °C) and only small differences between average nest and ambient temperatures (i.e. smaller than two standard deviations (SD) from the dataset’s average). These patterns indicated the female’s absence or low-quality data and those nights were, therefore, excluded. If available, we used both the previous and subsequent night to set thresholds for a given day. Then, the smaller values were used to pick the more sensitive thresholds for warming and less sensitive ones for cooling. Additional thresholds and all criteria were based on the method developed in Capilla-Lasheras (2018), and further determined by the evaluation of their fit to a subset of our data. This subset comprised diverse types of data, i.e. of different years, breeding stages, data quality and deserted broods.

*1.3 Assigning on- and off-bouts*

To assign on- and off-bouts to the single datapoints we followed five subsequent steps: We firstly assigned off-bouts to very low nest temperatures that follow the same pattern as ambient temperature (i.e. with an absolute difference between nest and ambient temperature of < 5 °C and absolute changes of these differences of < 0.5 °C). Secondly, we assigned on- and off-bouts to cumulative cooling and warming when the maximal temperature difference of the bout was twice as large as the average observed at daytime and when the cumulative change exceeded the maximum drop or rise at night-time. Thirdly, large temperature changes during daytime were determined as off- and on-bout when they exceeded the corresponding night-time maximal drop and rise, respectively (as in Capilla-Lasheras 2018). As a fourth step, presence of the female was assumed for nest temperatures increases if cooling would be expected otherwise from large differences to ambient temperature (i.e. at nest temperatures > 10 °C warmer than ambient temperatures at daytime and > 15 °C during night-time; equivalent to the *temp.diff.threshold* setting in Capilla-Lasheras, 2018). Lastly, standalone drops and rises were filtered automatically to be strong enough to be plausible for the 3-min interval. We considered an off-bout reasonable if the drop was equal to or lower than the corresponding night-time reference, and considered an on-bout reasonable if the rise was equal to or above the corresponding reference and the nest temperature did not deviate more than 15 °C from ambient temperature). For further details on the data processing see Strauß et al. (2024).

*1.4 Manual checking and assignment of quality*

To determine the chronotype for each female, we used the onset of activity, here determined as the first off-bout of the female from the nest after night-time. All data was visually inspected, and its quality was evaluated (very good (n = 2510), intermediate (n = 1203) and bad (n = 324)). One observer (AFTS) assessed the quality based on the visibility of the differences between active and inactive phase, i.e. high *versus* low nest temperature variation, respectively (Smith et al. 2015; Capilla-Lasheras 2018; Diez‐Méndez et al. 2021). Additionally, lower quality scores were given to low absolute nocturnal nest temperatures (medium: ca 15-20 °C; bad: < 15 °C) because of the suboptimal position of the temperature logger assumed for these cases. For this study we used very good onset data only. In 2% of the cases, where the on- and off-bout analytics were clearly too sensitive to nest temperature changes, the onset of activity was manually adjusted (to be earlier: n=3 or later: n=52).

All data processing and statistical analyses were performed in R (version 4.3.1; R Core Team 2023) and Rstudio (version 2023.06.2).

## 2. Full Estimates and Statistics of Repeatability, Fitness Parameters and Life History Traits

#### *Table S1: Sample size table – Inclusion and exclusion factors for data processing and statistical models*.

| **Analyses** | **Sample size** | **Inclusion or exclusion factor** |
| --- | --- | --- |
| Standardisation | 2512 onsets | Detectable onsets, excluding experimental days (Strauß et al. 2024), with at least 3 females measured per recorded date |
| Repeatability | 2316 standardised onsets of 184 females | Repeated measurements of known females |
| **Chronotype** | **164 females** | **One mean per female**; excluding experimental females (Strauß et al. 2024) |
| Clutch size & lay date | 164 females | none |
| Second brood | 131 females | Only females with successful first brood |
| Number of hatchlings & fledglings | 159 females | Excluding females with brood failure due to iB change (– 5 females) |
| Fledge success | 157 females | Excluding no-hatch females (– 2 females) |
| Female condition | 153 females | Depending on biometrics availability |
| Nestling condition | weight: 845 nestlings of 141 females | Depending on biometrics availability |
|  | tarsus: 682 nestlings of 119 females |  |
|  | P3: 835 nestlings of  140 females |  |

#### Table S2: Model estimates and statistics for the standardised activity onset. April day represents the day of the season, where April 1^st^ = 0, and Breeding day was centred April day relative to hatch date (i.e., hatch day = 0). P-values and statistics were assessed by stepwise reduction of the model and likelihood ratio testing. Estimates (Est) and standard errors (SEM) are provided for covariates at the time of dropping.

|  | |  | **Onset relative to Conspecifics** *Gaussian* | | | | |
| --- | --- | --- | --- | --- | --- | --- | --- |
|  | |  | n = 2316 of 184 females | | | | |
|  | | **df** | **Est** | **SEM** | **X^2^** | **p** |  |
| **Intercept** | 2020 |  | 0.034 | 0.043 |  |  |  |
| **Breeding day** | | 1 | -0.037 | 0.006 | 38.943 | <0.001 | *** |
| **Breeding day²** | | 1 | -0.004 | 0.001 | 54.533 | <0.001 | *** |
| **Year** | 2021 | 2 | 0.111 | 0.081 | 3.008 | 0.222 |  |
|  | 2022 |  | -0.001 | 0.090 |  |  |  |
| **April day** | | 1 | 0.002 | 0.004 | 0.184 | 0.668 |  |
| **April day: Year** | 2021 | 2 | -0.001 | 0.006 | 0.494 | 0.781 |  |
|  | 2022 |  | 0.004 | 0.008 |  |  |  |
|  | |  | **Var** | **SD** |  |  |  |
| **Female** | | 1 | 0.135 | 0.367 | 10.444 | 0.001 | ** |
| **Female_Year** | | 1 | 0.062 | 0.248 | 9.654 | 0.002 | ** |
| **Nest-box** | | 1 | 0.000 | 0.000 | 0.000 | 1.000 |  |
| **Residual** | |  | 0.737 | 0.858 |  |  |  |

#### Table S3: Model estimates and statistics for the annual brood success traits. Lay date was centred within year. P-values and statistics were assessed by stepwise reduction of the model and likelihood ratio testing. Estimates (Est) and standard errors (SEM) are provided for covariates at the time of dropping. Binomial model estimates are shown as average change in the log odds.

|  | |  | **Fledge success** *Binomial* n = 157 | | | | | | **Number of fledglings** *Gaussian* n = 159 | | | | | | **Number of hatchlings** *Gaussian* n = 159 | | | | | | |
| --- | --- | --- | --- | --- | --- | --- | --- | --- | --- | --- | --- | --- | --- | --- | --- | --- | --- | --- | --- | --- | --- |
|  | | **df** | **Est** | **SEM** | **Deviance** |  | **p** |  | **Est** | **SEM** | **Deviance** | **F** | **p** |  | **Est** | **SEM** | **Deviance** | **F** | **p** |  |  |
| **Intercept** | 2020 |  | 3.437 | 0.787 |  |  |  |  | 5.553 | 0.396 |  |  |  |  | 7.728 | 0.220 |  |  |  |  |  |
| **Chronotype** | | 1 | -0.939 | 0.633 | -2.233 |  | 0.135 |  | -0.373 | 0.424 | -5.728 | 0.775 | 0.380 |  | -0.158 | 0.433 | -1.026 | 0.133 | 0.716 |  |  |
| **Chronotype²** | | 1 | 0.828 | 1.277 | -0.451 |  | 0.502 |  | -0.967 | 0.682 | -14.753 | 2.009 | 0.158 |  | 0.193 | 0.709 | -0.573 | 0.074 | 0.786 |  |  |
| **Year** | 2021 | 2 | -1.532 | 0.810 | -8.205 |  | 0.017 | * | -1.453 | 0.520 | -251.140 | 17.013 | <0.001 | *** | 0.649 | 0.530 | -19.980 | 1.305 | 0.274 |  |  |
|  | 2022 |  | 0.566 | 1.258 |  |  |  |  | 1.575 | 0.560 |  |  |  |  | 0.883 | 0.571 |  |  |  |  |  |
| **Chronotype: Year** | 2021 | 2 | 3.319 | 2.278 | -2.806 |  | 0.246 |  | 0.168 | 1.087 | -13.520 | 0.916 | 0.402 |  | -0.453 | 1.119 | -3.371 | 0.215 | 0.807 |  |  |
|  | 2022 |  | 2.135 | 3.290 |  |  |  |  | 1.367 | 1.142 |  |  |  |  | 0.233 | 1.176 |  |  |  |  |  |
| **Chronotype²: Year** | 2021 | 2 | 3.104 | 3.421 | -9.999 |  | 0.007 | † | 1.776 | 1.941 | -24.500 | 1.675 | 0.191 |  | 1.480 | 2.017 | -5.1159 | 0.324 | 0.724 |  |  |
|  | 2022 |  | 556900.000 | 1384000.000 |  |  |  |  | -1.356 | 1.818 |  |  |  |  | 1.320 | 1.890 |  |  |  |  |  |
| **Lay date centred** | | 1 | 0.127 | 0.056 | -6.811 |  | 0.009 | ** | 0.020 | 0.030 | -3.487 | 0.473 | 0.493 |  | -0.140 | 0.030 | -169.120 | 22.002 | <0.001 | *** |  |

† Significant reduction of the model fit due to overfitting in the full model probably caused by the very few failed broods in 2020 (3 failed) and 2022 (1 failed) compared to 2021 (10 failed): model statistics for this interaction were non-significant per year (Chronotype^2^: Year 2021: z = 0.908, p = 0.364, and Chronotype^2^: Year 2022: z = 0.402, p = 0.687).

#### Table S4: Model estimates and statistics for nestling biometrics. Hatch date was centred within year. Nestling age is equivalent to chick day (= 0 at hatch day). For Time of day, we used a numeric count from midnight in hours. P-values and statistics were assessed by stepwise reduction of the model and likelihood ratio testing. Estimates (Est) and standard errors (SEM) are provided for covariates at the time of dropping.

|  | |  | **Nestling weight [g]** *Gaussian* | | | | | | **Nestling tarsus [mm]** *Gaussian* | | | | | | **Nestling P3 feather [mm]** *Gaussian* | | | | | |
| --- | --- | --- | --- | --- | --- | --- | --- | --- | --- | --- | --- | --- | --- | --- | --- | --- | --- | --- | --- | --- |
|  | |  | n = 845 of 141 females | | | | | | n = 682 of 119 females | | | | | | n = 835 of 140 females | | | | | |
|  | | **df** | **Est** | **SEM** | **X^2^** | **p** |  | **Est** | | **SEM** | **X^2^** | **p** |  | **Est** | | **SEM** | **X^2^** | **p** |  |  |
| **Intercept** | 2020 |  | -11.391 | 5.824 |  |  |  | 100.659 | | 24.923 |  |  |  | -151.146 | | 142.474 |  |  |  |  |
| **Chronotype** | | 1 | -0.025 | 0.247 | 0.013 | 0.911 |  | -1.517 | | 1.203 | 1.682 | 0.195 |  | -4.362 | | 6.019 | 0.556 | 0.456 |  |  |
| **Chronotype²** | | 1 | -0.171 | 0.435 | 0.167 | 0.683 |  | 0.217 | | 2.247 | 0.009 | 0.925 |  | 3.176 | | 10.581 | 0.096 | 0.757 |  |  |
| **Year** | 2021 | 2 | -0.301 | 0.300 | 18.595 | <0.001 | *** | -2.803 | | 1.401 | 10.312 | 0.006 | ** | -23.833 | | 7.932 | 12.389 | 0.002 | ** |  |
|  | 2022 |  | 1.079 | 0.315 |  |  |  | 2.999 | | 1.648 |  |  |  | 5.939 | | 7.852 |  |  |  |  |
| **Chronotype:**  **Year** | 2021 | 2 | 0.559 | 0.611 | 1.105 | 0.576 |  | 1.817 | | 2.638 | 0.541 | 0.763 |  | -1.313 | | 14.997 | 0.123 | 0.941 |  |  |
|  | 2022 |  | 0.093 | 0.638 |  |  |  | 0.534 | | 3.791 |  |  |  | 3.486 | | 15.708 |  |  |  |  |
| **Chronotype²:**  **Year** | 2021 | 2 | 0.034 | 1.130 | 0.369 | 0.832 |  | -0.538 | | 4.833 | 0.707 | 0.702 |  | -17.459 | | 27.465 | 0.786 | 0.675 |  |  |
|  | 2022 |  | 0.553 | 1.087 |  |  |  | 5.183 | | 7.319 |  |  |  | 4.052 | | 26.227 |  |  |  |  |
| **Hatch date centred** | | 1 | 0.005 | 0.012 | 0.189 | 0.664 |  | 0.183 | | 0.052 | 12.114 | 0.001 | *** | 0.599 | | 0.294 | 4.295 | 0.038 | * |  |
| **Number of fledglings** | | 1 | 0.231 | 0.064 | 12.730 | <0.001 | *** | 1.524 | | 0.294 | 25.692 | <0.001 | *** | 8.050 | | 1.569 | 25.226 | <0.001 | *** |  |
| **Nestling age** | | 1 | 1.739 | 0.388 | 19.440 | <0.001 | *** | 5.447 | | 1.660 | 10.809 | 0.001 | ** | 28.490 | | 9.489 | 9.115 | 0.003 | ** |  |
| **Time of day** | | 1 | 0.057 | 0.050 | 1.375 | 0.241 |  | - | |  | - |  |  | - | |  | - |  |  |  |
| **Time of day²** | | 1 | 0.031 | 0.018 | 3.021 | 0.082 | . | - | |  | - |  |  | - | |  | - |  |  |  |
|  | |  | **Var** | **SD** |  |  |  | **Var** | | **SD** |  |  |  | **Var** | | **SD** |  |  |  |  |
| **BroodID** | | 1 | 1.798 | 1.341 | 329.860 | <0.001 | *** | 31.540 | | 5.616 | 202.750 | <0.001 | *** | 1103.500 | | 33.220 | 376.790 | <0.001 | *** |  |
| **Residuals** | |  | 1.461 | 1.209 |  |  |  | 33.430 | | 5.782 |  |  |  | 686.200 | | 26.190 |  |  |  |  |

#### Table S5: Model estimates and statistics for female weight. For Time of day, we used a numeric count from midnight in hours. P-values and statistics were assessed by stepwise reduction of the model and likelihood ratio testing. Estimates (Est) and standard errors (SEM) are provided for covariates at the time of dropping.

|  | |  | **Female weight [g]** *Gaussian* n = 153 | | | | | |
| --- | --- | --- | --- | --- | --- | --- | --- | --- |
|  | | **df** | **Est** | **SEM** | **Deviance** | **F** | **p** |  |
| **Intercept** | 2020 |  | 7.876 | 1.865 |  |  |  |  |
| **Chronotype** | | 1 | 0.111 | 0.107 | -0.497 | 1.078 | 0.301 |  |
| **Chronotype²** | | 1 | 0.061 | 0.175 | -0.056 | 0.120 | 0.730 |  |
| **Year** | 2021 | 2 | 0.357 | 0.133 | -4.685 | 4.991 | 0.008 | * |
|  | 2022 |  | -0.002 | 0.144 |  |  |  |  |
| **Chronotype:** | 2021 | 2 | 0.616 | 0.277 | -2.259 | 2.478 | 0.088 | . |
| **Year** | 2022 |  | 0.359 | 0.292 |  |  |  |  |
| **Chronotype²:** | 2021 | 2 | 0.557 | 0.492 | -0.657 | 0.718 | 0.489 |  |
| **Year** | 2022 |  | 0.446 | 0.457 |  |  |  |  |
| **Time** | | 1 | 0.041 | 0.021 | -1.677 | 3.637 | 0.058 | . |
| **Time²** | | 1 | 0.005 | 0.007 | -0.245 | 0.529 | 0.468 |  |
| **Tarsus [mm]** | | 1 | 0.049 | 0.010 | -12.369 | 26.358 | <0.001 | *** |

#### Table S6: Model estimates and statistics for life-history traits, including lay date (April day of first egg), size of the first clutch, and initiation of a second brood. Lay date was centred within year for the models on clutch size and second brood. First brood success refers to the number of fledglings of the first clutch. First brood success reflects the number of fledglings of the first successful brood. P-values and statistics were assessed by stepwise reduction of the model and likelihood ratio testing. Estimates (Est) and standard errors (SEM) are provided for covariates at the time of dropping. Binomial model estimates are shown as average change in the log odds.

|  | |  | **Lay date** *Gaussian* n = 164 | | | | | | **Clutch size** *Gaussian* n = 164 | | | | | | | **Second brood** *Binomial* n = 131 | | | | | |
| --- | --- | --- | --- | --- | --- | --- | --- | --- | --- | --- | --- | --- | --- | --- | --- | --- | --- | --- | --- | --- | --- |
|  | | **df** | **Est** | **SEM** | **Deviance** | **F** | **p** |  | | **Est** | **SEM** | **Deviance** | **F** | **p** |  | **Est** | **SEM** | **Deviance** |  | **p** |  |
| **Intercept** | 2020 |  | 19.670 | 1.135 |  |  |  |  | | 7.720 | 0.112 |  |  |  |  | -2.001 | 0.297 |  |  |  |  |
| **Chronotype** | | 1 | 0.239 | 1.146 | -2.351 | 0.044 | 0.835 |  | | 0.091 | 0.223 | -0.347 | 0.166 | 0.684 |  | 0.418 | 0.575 | -0.533 |  | 0.465 |  |
| **Chronotype²** | | 1 | 4.095 | 1.821 | -273.320 | 5.058 | 0.026 | * | | -0.186 | 0.364 | -0.546 | 0.261 | 0.610 |  | 0.039 | 1.109 | -0.001 |  | 0.972 |  |
| **Year** | 2021 | 2 | 5.821 | 1.388 | -2492.300 | 23.059 | <0.001 | *** | | 0.091 | 0.271 | -1.776 | 0.428 | 0.653 |  | 1.709 | 0.853 | -5.568 |  | 0.062 | . |
|  | 2022 |  | -3.404 | 1.518 |  |  |  |  |  | 0.268 | 0.296 |  |  |  |  | 1.583 | 0.886 |  |  |  |  |
| **Chronotype:** | 2021 | 2 | 0.030 | 2.933 | -0.030 | 0.000 | 1.000 |  | | -0.008 | 0.570 | -8.599 | 2.079 | 0.129 |  | -2.795 | 2.163 | -2.838 |  | 0.242 |  |
| **Year** | 2022 |  | -0.036 | 3.106 |  |  |  |  |  | 1.010 | 0.604 |  |  |  |  | -0.961 | 2.147 |  |  |  |  |
| **Chronotype²:** | 2021 | 2 | 11.139 | 5.111 | -255.600 | 2.376 | 0.096 | . | | 0.678 | 1.015 | -5.890 | 1.432 | 0.242 |  | 1.415 | 6.447 | -0.801 |  | 0.670 |  |
| **Year** | 2022 |  | 6.429 | 4.895 |  |  |  |  |  | -0.820 | 0.963 |  |  |  |  | 3.390 | 6.176 |  |  |  |  |
| **Lay date centred** | | 1 | - |  | - |  |  |  | | -0.055 | 0.015 | -27.175 | 13.184 | <0.001 | *** | -0.135 | 0.050 | -9.128 |  | 0.003 | ** |
| **First brood success** | | 1 | - |  | - |  |  |  | | - |  | - |  |  |  | 0.140 | 0.168 | -0.720 |  | 0.396 |  |

## 3. Re-analyses of other datasets using standardised chronotype

Onset of activity from three freely available datasets (Graham et al. 2017; Meijdam et al. 2022; Womack et al. 2023) were standardised within date to match our chronotype proxy, and analysed in the same manner as described in the Methods of the manuscript. The data from Graham et al. and Meijdam et al. only comprised one year each. Thus, *Year* and *Chronotype* interactions with year were excluded from the statistics.

#### Table S7: Model estimates and statistics for the number of fledglings and lay date using standardised chronotype of Womack et al. Data from forest areas only, and lay date was estimated by the subtraction of clutch size from incubation start date plus 1. For the fledgling model, lay date was centred within year. P-values and statistics were assessed by stepwise reduction of the model and likelihood ratio testing. Estimates (Est) and standard errors (SEM) are provided for covariates at the time of dropping.

| **Womack** n = 60 | |  | **Number of fledglings** | | | | | | **Lay date** | | | | | |
| --- | --- | --- | --- | --- | --- | --- | --- | --- | --- | --- | --- | --- | --- | --- |
|  | | **df** | **Est** | **SEM** | **Deviance** | **F** | **p** |  | **Est** | **SEM** | **Deviance** | **F** | **p** |  |
| **Intercept** | 2016 |  | 5.000 | 0.315 |  |  |  |  | 31.913 | 0.904 |  |  |  |  |
| **Chronotype** | | 1 | -0.722 | 0.543 | -10.374 | 1.768 | 0.189 |  | -0.098 | 0.980 | -0.193 | 0.010 | 0.920 |  |
| **Chronotype²** | | 1 | 0.838 | 1.127 | -3.265 | 0.552 | 0.461 |  | 1.581 | 2.023 | -11.760 | 0.611 | 0.438 |  |
| **Years** | 2017 | 2 | -0.979 | 0.681 | -12.438 | 1.046 | 0.358 |  | -2.430 | 1.210 | -176.791 | 4.705 | 0.013 | ***** |
|  | 2018 |  | -0.690 | 1.001 |  |  |  |  | 2.462 | 1.779 |  |  |  |  |
| **Chronotype:** | 2017 | 2 | -0.820 | 1.192 | -5.226 | 0.432 | 0.651 |  | 2.779 | 2.097 | -34.542 | 0.894 | 0.415 |  |
| **Years** | 2018 |  | -1.735 | 2.133 |  |  |  |  | 2.206 | 3.802 |  |  |  |  |
| **Chronotype²:** | 2017 | 2 | -0.076 | 2.670 | -1.571 | 0.126 | 0.882 |  | -0.415 | 4.778 | -2.654 | 0.066 | 0.936 |  |
| **Years** | 2018 |  | 5.411 | 11.101 |  |  |  |  | 6.597 | 19.848 |  |  |  |  |
| **Lay date centred** | | 1 | -0.245 | 0.075 | -64.440 | 10.816 | 0.002 | ** | - |  | - |  |  |  |

#### Table S8: Model statistics for lay date (April day of first egg) using standardised chronotype of Graham et al. and Meijdam et al. datasets which comprised only one year of data. P-values and statistics were assessed by stepwise reduction of the model and likelihood ratio testing. Estimates (Est) and standard errors (SEM) are provided for covariates at the time of dropping.

| **Lay date** |  | **Graham** n = 13 | | | | | | **Meijdam** n = 138 | | | | | |
| --- | --- | --- | --- | --- | --- | --- | --- | --- | --- | --- | --- | --- | --- |
|  | **df** | **Est** | **SEM** | **Deviance** | **F** | **p** |  | **Est** | **SEM** | **Deviance** | **F** | **p** |  |
| **Intercept** |  | 13.385 | 0.805 |  |  |  |  | 7.884 | 0.510 |  |  |  |  |
| **Chronotype** | 1 | 1.151 | 1.320 | -6.529 | 0.760 | 0.402 |  | -0.341 | 0.642 | -10.152 | 0.282 | 0.597 |  |
| **Chronotype²** | 1 | -1.007 | 1.557 | -3.791 | 0.418 | 0.533 |  | 0.882 | 0.568 | -86.129 | 2.413 | 0.123 |  |
